# Effect of CRM team leader training on team performance and leadership behavior in simulated cardiac arrest scenarios: a prospective, randomized, controlled study

**DOI:** 10.1186/s12909-015-0389-z

**Published:** 2015-07-24

**Authors:** Ezequiel Fernandez Castelao, Margarete Boos, Christiane Ringer, Christoph Eich, Sebastian G. Russo

**Affiliations:** 1 Department of Social and Communication Psychology, Georg-Elias-Müller Institute of Psychology, Georg-August-University Göttingen, Gosslerstraße 14, 37073 Göttingen, Germany; 2 Department of Anaesthesiology, University Medical Centre Göttingen, Robert-Koch-Straße 40, 37075 Göttingen, Germany; 3 Department of Anaesthesia, Paediatric Intensive Care and Emergency Medicine, Auf der Bult Children’s Hospital, Janusz-Korczak-Allee 12, 30173 Hannover, Germany

## Abstract

**Background:**

Effective team leadership in cardiopulmonary resuscitation (CPR) is well recognized as a crucial factor influencing performance. Generally, leadership training focuses on task requirements for leading as well as non-leading team members. We provided crisis resource management (CRM) training only for designated team leaders of advanced life support (ALS) trained teams. This study assessed the impact of the CRM team leader training on CPR performance and team leader verbalization.

**Methods:**

Forty-five teams of four members each were randomly assigned to one of two study groups: CRM team leader training (CRM-TL) and additional ALS-training (ALS add-on). After an initial lecture and three ALS skill training tutorials (basic life support, airway management and rhythm recognition/defibrillation) of 90-min each, one member of each team was randomly assigned to act as the team leader in the upcoming CPR simulation. Team leaders of the CRM-TL groups attended a 90-min CRM-TL training. All other participants received an additional 90-min ALS skill training. A simulated CPR scenario was videotaped and analyzed regarding no-flow time (NFT) percentage, adherence to the European Resuscitation Council 2010 ALS algorithm (ADH), and type and rate of team leader verbalizations (TLV).

**Results:**

CRM-TL teams showed shorter, albeit statistically insignificant, NFT rates compared to ALS-Add teams (mean difference 1.34 (95 % CI −2.5, 5.2), *p* = 0.48). ADH scores in the CRM-TL group were significantly higher (difference −6.4 (95 % CI −10.3, −2.4), *p* = 0.002). Significantly higher TLV proportions were found for the CRM-TL group: direct orders (difference −1.82 (95 % CI −2.4, −1.2), *p* < 0.001); undirected orders (difference −1.82 (95 % CI −2.8, −0.9), *p* < 0.001); planning (difference −0.27 (95 % CI −0.5, −0.05) *p* = 0.018) and task assignments (difference −0.09 (95 % CI −0.2, −0.01), *p* = 0.023).

**Conclusion:**

Training only the designated team leaders in CRM improves performance of the entire team, in particular guideline adherence and team leader behavior. Emphasis on training of team leader behavior appears to be beneficial in resuscitation and emergency medical course performance.

**Electronic supplementary material:**

The online version of this article (doi:10.1186/s12909-015-0389-z) contains supplementary material, which is available to authorized users.

## Background

In cardiopulmonary resuscitation (CPR), effective leadership is positively associated with patient outcome [1–4]. Therefore, concepts and methods for training team leaders to effectively steer and coordinate resuscitation teams are under investigation [5–9]. Moreover, both the European Resuscitation Council (ERC) and the American Heart Association (AHA) strongly recommend integrating teamwork training, including leadership as a key skill, into advanced life support (ALS) education [10, 11].

Regarding the implemented teaching methods, some training approaches combine patient simulation with debriefings on team and leadership behavior [7, 9, 12–14]. Other approaches are less extensive (i.e., no debriefings) and some comprise more invasive teaching elements such as brief instructions [8, 15, 16]. However, the inclusion of team leadership behavior as a main topic in CPR training has been positively evaluated by many studies, showing short- and long-term positive effects on CPR performance [8, 16–20]. Such training approaches have been designed to impart specific knowledge on how leaders can contribute systematically to team effectiveness in CPR. According to the model of functional team leadership, team effectiveness is determined by a reciprocal influence between the effects the four primary leadership functions (information search and structuring, problem solving, managing personnel resources, and managing material resources) and the four types of team processes (cognitive, motivational, affective, and coordination) [21]. Depending on the experience and skills of leader(s) and team members and on the context factors of the task at hand, the complexity of such a multifactorial structure increases, creating the potential for an incomplete understanding of the whole process. This incomplete understanding is even more likely during CPR training of inexperienced trainees (e.g., graduates, junior physicians) required to function in the typical CPR setting characterized by time pressure and imminent danger to the patient’s life. Thus, reducing the cognitive workload beforehand by separating role instructions can have a positive impact on the CPR performance and on the trainees’ individual learning process [22, 23].

Team-oriented trainings focus on the behavior of the entire team, emphasizing that *all* trainees acquire knowledge about the task requirements of team leaders *and* non-leading team members. In clinical reality, however, teams are usually assembled as ad hoc “crews,” making it unrealistic to establish team mental models as an enduring common cognitive structure [24–26]. Furthermore, CPR as a task is highly predefined by the ERC guidelines. One of the main purposes of these guidelines is so the accomplishment of subtasks and their sequences do not necessarily have to be discussed in the team setting; all team members have a pre-task cognitive understanding of what needs to be accomplished when performing CPR. Based on these conditions, it is not absolutely necessary to train how to function as a leader and as a non-leading team member in a CPR situation at the same time. In order to achieve a more sustainable learning effect by stepwise learning role requirements, we argue that it is more reasonable to separate trainings for leader and non-leader team members.

In this study we provided crisis resource management (CRM) training for a single team member only in order to qualify him or her as the team leader. We hypothesized that ALS-trained teams with a CRM-trained leader show (1) higher adherence to the ALS guidelines (ADH), (2) lower no-flow time (NFT), and (3) higher quality of team leaders’ verbalizations (TLV), as compared to teams lead by leaders without CRM-training. Furthermore, we hypothesized (4) a positive link between CPR outcomes — ADH and NFT — and team leaders’ verbalizations. The objective of our study was therefore to determine whether said CRM training for a single team member only is linked to positive CPR outcomes and team leader verbalization.

## Methods

This randomized, controlled, simulator-based study was embedded in two independent editions of a two-week mandatory course for fifth-year medical students at the Georg-August University Medical School in Göttingen (Germany) covering intensive care and emergency medicine. The first edition of the course took place in December 2010 and the second in May 2011. The core components regarding basic and advanced life support (BLS and ALS) consisted of a 90-min lecture and four instructor-led interactive tutorials: (1) BLS, (2) airway management, (3) rhythm recognition/defibrillation, and (4) a team action simulated CPR scenario, videotaped to facilitate later evaluation.

The study was approved by the Ethics Committee of Georg-Elias-Müller Institute of Psychology in Göttingen, Germany (09–2011). All participating students provided written permission prior to being videotaped during the simulated CPR scenario (the fourth and final tutorial).

### Study participants and design

**Fig. 1:**
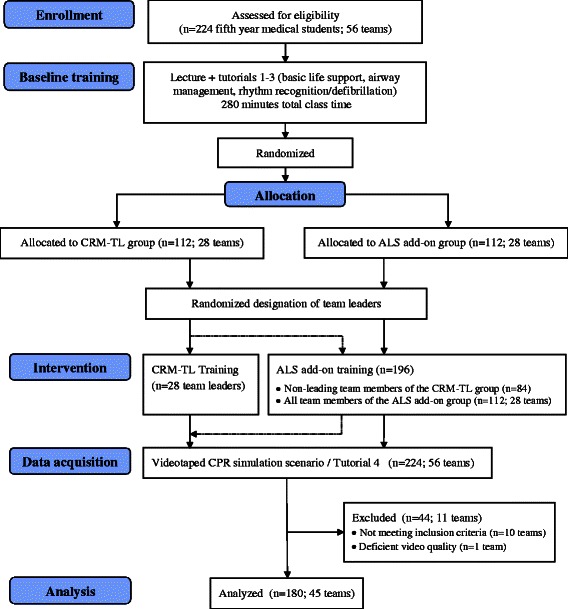
Flow Diagram of study design and sample sizes (Consort). Dotted line represents non-leading members of teams allocated to the CRM-TL group. They completed ALS add-on training before data acquisition

Two-hundred and twenty-four (224) students were quasi-randomly (alphabetically) assigned to 56 teams of four students each. After attending the 90-min lecture as well as the first three tutorials described in the above paragraph, the 56 teams were randomly allocated to one of two groups: (group 1) the CRM team leader training (CRM-TL) group or (group 2) the additional ALS training (ALS add-on) group. In both groups, one person from each team was randomly designated to be the team leader in the upcoming CPR simulation, the difference being that the 28 designated team leaders from the CRM-TL group (group 1) attended the CRM-TL training. The non-leading team members of the teams allocated to the CRM-TL group (group 1) and all members of the teams allocated to the ALS add-on group (group 2) attended the additional ALS training (see Fig. 1 showing the design, procedure, and sample sizes of the study).

In Tutorial 4, all teams had to master the same standardized CPR scenario. For educational and ethical reasons, after the simulation all students were given whichever training had been temporarily excluded for purposes of this study. To avoid knowledge bias, we excluded teams having members with pertinent emergency care experience or incomplete attendance at prior lectures and/or tutorials. In order to protect the privacy of student attendance information, allocation concealment of students’ lecture and tutorial attendance was documented by teaching staff.

### Training concepts

The 90-min CRM-TL training attended by the 24 designated team leaders from the CRM-TL group—subdivided in three groups of eight students—was a slightly modified version of the CRM team training for CPR treatments, which was evaluated in a previous study from our group [18]. It consisted of a presentation on the theory of CRM, the integration of CRM into CPR, and three video presentations showing teams performing ALS combined with an oral exercise to assure knowledge transfer. During the whole training students were explicitly invited to continuously reflect, discuss, and challenge topics in order to facilitate active participation and enable concurrent feedback from the teaching staff. In this modified version of the training, the focus was on the team leader with regard to coordination and time management as well as explicit methods (i.e., verbalizations) of communication and leadership.

The additional ALS training that the condition group attended also took 90 min and consisted of a summary of the initial BLS/ALS lecture and the first three interactive tutorials covering basic life support, airway management, and rhythm recognition/defibrillation but did not include the team leadership issues covered in the CRM-TL training version.

### Scenario and acquisition of data

The CPR scenario used for this study was designed as a monitored simulation tutorial to apply and discuss all previously learned ALS treatment elements. It took place in the training and simulation center for students (STÄPS) at the Georg-August University Medical School and was videotaped for later analysis. A resuscitation manikin (ResusciAnne®, Laerdal, Stavanger, Norway), lying on the floor in the middle of the training area, enabled realistic chest compression and ventilation.

The simulated scenario was a witnessed cardiac arrest at the ward of a large district hospital. The resuscitation team was summoned by a ward nurse and had immediate access to standard ALS equipment. The task of the resuscitation team was to apply ALS that adhered to the 2010 ERC guidelines until the return of spontaneous circulation (ROSC) [27]. In cases of successful application of the algorithm, the instructor ended the simulation after the fourth defibrillation. If a team failed to defibrillate the patient four times within 15 min, the instructor was obligated to end the simulation. At the end of the tutorial the students were asked to complete a questionnaire with demographic data.

### Outcome measures and assessment

Our primary endpoints were NFT and ADH as the two categories regarding quality of CPR as well as TLV as the crucial dimension of communication.

Each scenario was analyzed until the fourth defibrillation, using the coding software Interact 9 [28]. NFT was defined as the percentage of the scenario time frame minus the time with chest compressions performed by any team member. To determine ADH, a panel of emergency medicine experts developed a checklist-based tool to evaluate the key components of CPR according to the 2010 ERC guidelines (see Additional file 1). The checklist was Delphi-validated and consisted of 13 items, each weighted according to the experts’ importance ratings. The assessment was carried out by one of the authors (CR) blinded to the experimental conditions. She viewed the video recordings to determine the teams’ guidelines adherence following the aforementioned checklist. To confirm the correctness of the rating procedure, the results were discussed with another author (SR), also blinded to the experimental allocation.

To assess TLV, we focused on four categories of a taxonomy we applied in a previous evaluation study from our group. It consisted of 44 observable utterances and task relevant actions indexed by leadership statements, different types of questions, coordination mechanisms, CPR treatment related actions [18]. Each observable verbalization that could be classified into one of the categories was documented, including communicator or actor. We trained four coders for a total of 15 h and reached substantial inter-rater reliability (κ = 0.61).

The four categories we focused on were: (1) *direct orders* or personally addressed demands were single tasks assigned to a specific team member; the addressee must be distinctly addressed by name, physical gesture or eye contact. (2) *Undirected orders* were all other demands not fulfilling the conditions of (1). (3) *Planning* behaviors were verbalizations containing at least two concrete actions to be provided within the CPR process, informing team members of upcoming or desired actions and not necessarily addressed to a specific team member. (4) *Task assignments* were direct orders containing the assignment of at least two different tasks. All four verbalization categories had a common key function: coordination mechanisms whose rate of occurrence was positively linked to teamwork quality [2, 29]. Table 1 offers observed examples from our data for each category.

**Table 1:** Team leader verbalization examples and occurrences^a^

Category	Examples	Group	
		CRM-TL-training (*n* = 24)	ALS add-on training (*n* = 2)
Direct orders	“Daniel^b^, please begin with chest compressions”	14.54 (7.3)	3.90 (3.6)
Undirected orders	“Give adrenaline, now” (Without physical or eye contact)	25.58 (10.8)	16.33 (9.1)
Planning	“Let’s provide two more 30–2 cycles. Then, we’ll reevaluate the rhythm”	2.83 (2.9)	1.52 (2.4)
Task assignments	“Linda^b^, please replace Daniel; Steve drew up adrenaline”	0.66 (0.9)	0.24 (0.5)

^a^Data are means (SD)

^b^Fictitious name

### Statistical analysis

The data were analyzed using SPSS 20 program (SPSS Inc., Chicago, IL, USA). Descriptive statistical analysis was performed for NFT, ADH, and TLV. Statistical significance (*p* < 0.05) of NFT and ADH scores of CRM-TL compared to ALS add-on groups was tested by *t*-tests (95 % CI) for independent samples. Intra-class correlation coefficients (ICC) were calculated for all variables.

## Results

### Flow and baseline characteristics

A total of 45 four-person teams consisting of 180 fifth-year medical students met the study inclusion criteria in December 2010 and May 2011. In terms of age (mean difference −0.6 (95 % CI, −1.3, 0.1), *p* = 0.11), gender (mean difference −4.8 (95 % CI −18.9, 9.4), *p* = 0.5) and familiarity (mean difference −0.1 (95 % CI −0.5, 0.3), *p* = 0.6) the groups were well balanced (Tables 2 and 3). Ten teams had to be excluded as at least one team member in each of these teams had been non-complaint with attending tutorials or lectures. Another team had to be excluded due to video camera malfunction. Of the remaining 45 teams, 24 had been previously randomly allocated to the CRM-TL training group and 21 to the ALS add-on group. Average CPR simulation duration was 694.67 s (±112.61).

**Table 2:** Team baseline demographic^a^

	Group	
	CRM-TL training (*N* = 24)	ALS add-on training (*N* = 21)
Sex (female)	56^b^ (58.33 %)	45^b^ (53.57 %)
Age (years)	26.09 (1.59)	25.49 (0.79)
Team familiarity (6 = high, 1 = low)	5.48 (0.75)	5.36 (0.71)
CPR scenario duration (seconds)	656 (99)	73 (113)

^a^Data are means (SD) or numbers (%)

^b^Number of individuals

**Table 3:** Team leader baseline demographic^a^

	Group	
	CRM-TL training (*N* = 24)	ALS add-on training (*N* = 21)
Sex (female)	9 (37.5 %)	13 (61.9 %)
Age (years)	25.7 (1.48)	25.09 (2.04)
Team familiarity (6 = high, 1 = low)	5.41 (1.28)	5.19 (1.21)

^a^Data are means (SD) or numbers (%)

### No-flow time & ALS guidelines adherence

On average, teams showed shorter, albeit statistically non-significantly shorter, NFT percentage after CRM-TL training compared to the ALS add-on group (27.75 % (±6.09) vs. 29.09 % (±6.71), difference 1.34 (95 % CI −2.5, 5.2), *p* = 0.485). ADH scores in the CRM-TL group were significantly higher than in the ALS add-on group (37.58 points (±6.02) vs. 31.41 points (±7.06), difference −6.4 (95 % CI −10.3, −2.4), *p* = 0.002).

### Team leaders’ verbalization quality and its relation to CPR performance

On average, 776.76 (±280.06) coding units per team and 253.82 (±107.68) coding units per team leader were coded. Significantly higher proportions in all four TLV categories were found for teams of the CRM-TL group (Table 4): *direct orders* (2.29 % (±1.20) vs. 0.47 % (±0.40), difference −1.82 (95 % CI −2.4, −1.2), *p* < 0.001); *undirected orders* (3.90 % (±1.70) vs. 2.08 % (±1.46), −1.82 (95 % CI −2.8, −0.9), *p* < 0.001); *planning* (0.44 % (±0.47) vs. 0.17 % (±0.24), difference −0.27 (95 % CI −0.5, −0.05),*p* = 0.018) and *task assignments* (0.11 % (±0.16) vs. 0.02 % (±0.05), difference −0.09 (95 % CI −0.2, −0.01), *p* = 0.023).

**Table 4:** CPR performance and team leader verbalizations^a^

	CRM-TL training (*n* = 24)	ALS add-on training (*n* = 21)	Difference (95 % CI)	*p*
CPR performance measures				
No-flow time (%)	27.75 (6.09)	29.09 (6.71)	1.34 (95 % CI −2.5, 5.2)	0.485
Adherence to ALS Guidelines (60 = high, −37 = low)	37.58 (6.02)	31.41 (7.06)	−6.4 (95 % CI −10.3, −2.4)	0.002
Team leader verbalization categories^b^				
Direct orders (%)	2.29 (1.20)	0.47 (0.40)	−1.82 (95 % CI −2.4, −1.2)	<0.001
Undirected orders (%)	3.90 (1.70)	2.08 (1.46)	−1.82 (95 % CI −2.8, −0.9)	<0.001
Planning (%)	0.44 (0.47)	0.17 (0.24)	−0.27 (95 % CI −0.5, −0.05)	0.018
Task assignments (%)	0.11 (0.16)	0.02 (0.05)	−0.09 (95 % CI −0.2, −0.01)	0.023

^a^ Data are means (SD)

^b^ Proportions of total team verbalization

Regarding the expected link between TLV and the CPR performance, none of the observed categories were significantly related to either ADH or NFT (Table 5).

**Table 5:** Correlations between team leader verbalization and CPR performance outcomes

Variable	1	2	3	4	5	6	7
1. CRM-TL training	―						
2. NFT	−.249	―					
3. ADH	.444**	−.346*	―				
TLV							
4. Direct orders	.708**	−.219	.209	―			
5. Undirected orders	.505**	−.091	.092	.525*	―		
6. Planning	.342*	−.133	.137	.220	.124	―	
7. Task assignments	.328*	−.096	−.164	.274	.405**	.409**	―

* = *p* < 0.05, ** = *p* < 0.01; 95 % CI

## Discussion

This randomized controlled study was designed to evaluate the impact of an interactive CRM training focusing on team leaders only. We found that teams composed of one CRM-trained team leader and three ALS-trained team members showed higher ADH scores during simulated CPR scenarios as compared to teams with a non-CRM-trained team leader. Additionally, the CRM-trained team leaders showed higher proportions of high quality verbal behavior (TLV) than their ALS-only trained counterparts. Unexpectedly, NFT rates in did not differ significantly and high quality TLV and CPR performance were not significantly correlated.

That said, our data provide some evidence that one leadership-skilled, CRM-trained team leader suffices to improve the team’s CPR performance. This finding is in line with the results of previous evaluations of leadership trainings not only in the medical field [16, 20, 30] but also in other emergency team fields [31, 32]. To our knowledge this study is the first empirical assessment of how CRM training for only the team leaders influences the performance of the whole team.

The role of the team leader during CPR is known to be pivotal, as she or he is the person responsible for the distribution and coordination of subtasks, ideally using clear and explicit communication [2, 3, 21, 33]. Our results suggest that it is not just the role of a team leader and her/his training in managing the task technically that seems to be crucial for the whole team to perform well but rather explicit (i.e., CRM) training in how to manage the resources of a team in order to perform its task in a planned and well-coordinated way. This differentiation is crucial to the clinical reality of how CPR teams are assembled as ad hoc “crews,” where members bring different resources (skill-sets and experience levels) to the CPR task, as we discuss below.

The positive impact of our CRM team leader training on ADH can be explained by two crucial arguments. First, the workload of each team member is likely reduced by the explicitly predefined leadership role. Indeed, the non-leading CPR team member role, especially for non-experts, is time and cognitively challenging, particularly in the early phase of CPR treatment [17, 22, 34, 35]. Provided that the team roles are actually adopted and the team leader distributes and coordinated subtasks effectively, the non-leading team members can focus on the accomplishment of their assigned subtasks (chest compression, airway management, etc.), facilitating the prompt and accurate provision of ALS adhering to the guidelines. The team leader, on the other hand, can delegate the actual hands-on tasks, keeping his or her hands and mind free to coordinate the whole resuscitation process. For any team leader, especially one who is inexperienced, this is a demanding task, emphasizing the benefits from leadership training especially designed to impart theoretical and practical knowledge to cope with this situation (i.e., coordination and time management as well as explicit methods of communication and leadership).

Second, although our study found no significant correlation between CPR performance and TLV, we assume that the accuracy of team leaders’ utterances is of critical importance in determining CPR outcome, as shown in previous studies [16, 18]. In an exploratory simulator study, junior physicians partly failed to delegate and communicate effectively during CPR and subsequently asked for more leadership training, including practical recommendations for verbal behavior [36]. In our study, team leaders of the CRM-TL group showed significantly more effective verbalizations than the team leaders of the ALS add-on group. The question of which verbal behavior(s) serve(s) best as coordination (and motivation) mechanism(s) to improve CPR team performance should therefore be further investigated by analyzing not only single verbalization mechanisms but sequences of verbal behavior. Selectively focusing on closed-loop communication patterns between leading and non-leading team members may shed additional light on the CPR teamwork process [37, 38], as this differentiation has been found to discriminate higher- from lower-performing medical high-risk teams [39].

Practical implications of our findings may contribute to improving the curricula for emergency medicine, particularly for medical students in their final stage of undergraduate education. The team role concept of leaders trained in a separate explicit step appears to be an efficient way to foster team processes and outcomes as a whole. To be a team leader or a non-leading team member are not conceptualized as hierarchical roles but as roles defined by discrete behaviors that have to be applied according to the specific individual resources (skill sets and experience levels) brought to the CPR task at hand. Given that CPR as a task is explicitly predefined by the guidelines, the integration of separately trained CRM-leaders and skilled team members can be expected to be frictionless. The theoretical implications of our study are that we contribute to newer, clinically realistic conceptualizations of leadership and followership as role-dependent sets of specific functional behaviors to be enacted dynamically and often times shared within a changing CPR task setting [21].

Our study has shown that CRM training for team leaders only is more effective than mere clinical training of every team member. In sum, our results suggest that there are both outcome and human resource advantages to discretizing the education of CPR team leader and follower behavior, thus reducing the workload for both roles and avoiding role conflicts. Especially in clinical practice—when it is often required to define and maintain a leader in a multi-professional ad-hoc team during an unexpected situation—involved team members can benefit from being aware of the different role requirements.

This study has its limitations. Regarding study design, we did not apply pre-training measurements of the students’ CPR skills. Randomization should have addressed this but we cannot rule out that systematic differences in the students’ baseline skills did not impacted their later performance. Due to module restrictions of the University Medical School in Göttingen, students had to be quasi-randomly (alphabetically) assigned to four-person teams whereas teams were randomly allocated to groups. In order to keep unsystematic variation to a minimum, we also assessed familiarity level of team members, which resulted in no relevant differences. Furthermore, we were unable to calculate sample size as we were required to train all students. We were also unable to demonstrate the retention potential of our CRM-TL training as we had not yet executed a training follow-up measure. Finally, we only focused on the team leaders’ verbal behavior, without taking team followers’ verbalizations into account. Our aim was to focus on the leadership aspect of CPR, as it has been widely recognized as being linked to the adherence to establish protocols, fewer errors, and more favorable patient outcome [1, 3, 40, 41].

## Conclusions

Our results demonstrate that CRM training only for team leaders can enhance overall CPR performance by increasing the adherence to the ALS guidelines, improving the quality of team leadership, and relieving the cognitive burden of all team members—leaders and followers alike. The results also suggest advantages of considering the dissimilarities of leading and non-leading roles and their discrete demands during CPR, minimizing workload demands and role conflicts for both leader and non-leader team members. Thus, to separately teach CPR leadership is recommended in order to improve both learning and performance effects, especially in the early stages of emergency care education. Furthermore, the results of our research support and extend the ERC and AHA recommendation of combining clinical competencies with coordination and communication skills of CPR within medical education.

## Additional file

Additional file 1: **Algorithm adherence score taxonomy modeled after the 2010 ERC guidelines.** (DOCX 20 kb)

## Abbreviations

ADH: Adherence to the 2010 Advance life support guidelines
AHA: American Heart Association
ALS: Advance life support
ALS-add-on: Additional advance life support skill training
BLS: Basic life support
CPR: Cardiopulmonary resuscitation
CRM: Crisis resource management
CRM-TL: Crisis resource management training for team leader
ERC: European Resuscitation Council
NFT: No-flow time
TLV: Team leader verbalization
