# Supplementary material for: Effect of CRM team leader training on team performance and leadership behavior in simulated cardiac arrest scenarios: a prospective, randomized, controlled study
Source: BMC Med Educ. 2015 Jul 24;15:116. doi: 10.1186/s12909-015-0389-z (PMC4526177; doi:10.1186/s12909-015-0389-z)
Supplement: Additional file 1: — Algorithm adherence score taxonomy modeled after the 2010 ERC guidelines. (DOCX 20 kb) [file 12909_2015_389_MOESM1_ESM.docx]

| **Category** | **Sub-categories (clinical-actions)** | **Points** |  | **Sum** |
| --- | --- | --- | --- | --- |
| Initial assessment | Assessment of responsiveness  Check for breathing  Check for pulse/signs of life  Recognition and verbalisation of ‘cardiac arrest’ | 1  1  1  1 | *Max*  *Min* | *4*  *0* |
| Rhythm diagnosis | ECG leads attached and rhythm analysed within 1 min  Chest compressions while attaching ECG | 2  2 | *Max*  *Min* | *4*  *0* |
| 1^st^ defibrillation | No delay of defibrillation  Safety  Gel/gel pads applied  Chest compressions while charging  No chest compressions while charging | 4  1  1  2  -2 | *Max*  *Min* | *8*  *-2* |
| 2^nd^ defibrillation | CPR interval 2min  +/-15 sec  +/-30 sec  Safety  Chest compressions while charging  No chest compressions while charging | 2  1  1  2  -2 | *Max*  *Min* | *5*  *-2* |
| 3^rd^ defibrillation | CPR interval 2min  +/-15 sec  +/-30 sec  Safety  Chest compressions while charging  No chest compressions while charging | 2  1  1  2  -2 | *Max*  *Min* | *5*  *-2* |
| 4^th^ defibrillation | CPR interval 2min  +/-15 sec  +/-30 sec  Safety  Chest compressions while charging  No chest compressions while charging | 2  1  1  2  -2 | *Max*  *Min* | *5*  *-2* |
| i.v. access | Established | 1 | *Max*  *Min* | *1*  *0* |
| Medication – adrenaline | Administered  Not administered  Right timing (after 3^rd^ defibrillation)  Wrong timing  Repetitive administration every 3-5 mins | 1  -1  2  -3  1 | *Max*  *Min* | *4*  *-3* |
| Medication – amiodarone | Administered  Not administered  Right timing (after 3^rd^ defibrillation)  Wrong timing | 1  -1  2  -3 | *Max*  *Min* | *3*  *-3* |
| Intubation | Laryngoscopy while performing chest compressions  No chest compressions during laryngoscopy  Successful intubation within 10 sec  More than 10 sec for intubation  Verification of endotracheal tube placement  No verification of correct tube placement | 2  -2  1  -2  3  -3 | *Max*  *Min* | *6*  *-7* |
| Chest compressions / ventilation | 30:2 before intubation  Not synchronised before intubation  Continuous chest compressions after intubation  Still 30:2 after intubation | 2  -4  4  -4 | *Max*  *Min* | *6*  *-8* |
| After rhythm conversation (after 4^th^ defibrillation) | CPR after rhythm conversion 2min  +/-15 sec  +/-30 sec  No CPR after rhythm conversion | 2  1  -4 | *Max*  *Min* | *2*  *-4* |
| Evaluation after ROSC | Sinus rhythm detected  No detection of sinus rhythm  Check for carotid pulse  BP taken  Discussion of post-resuscitation care | 4  -4  1  1  1 | *Max*  *Min* | *7*  *-4* |
| **Total** |  |  | *Max*  *Min* | *60*  *-37* |

Appendix 1: Algorithm adherence score taxonomy modeled after the 2010 ERC guidelines.

*Note*: This checklist-based assessment was developed to rate the adherence to the guidelines on the basis of video recordings. Depending on their clinical relevance, each clinical action (sub-category) is equivalent to a specific number of points. The sum represents final adherence score.
